# Isolated Short Fetal Femur Length in the Second Trimester and the Association with Adverse Perinatal Outcome: Experiences from a Tertiary Referral Center

**DOI:** 10.1371/journal.pone.0128820

**Published:** 2015-06-05

**Authors:** Mariella Mailath-Pokorny, Stephan Polterauer, Katharina Worda, Stephanie Springer, Dieter Bettelheim

**Affiliations:** 1 Department of Obstetrics and Gynecology, Division of Obstetrics and fetomaternal Medicine, Medical University of Vienna, Vienna, Austria; 2 Department of Obstetrics and Gynecology, Division of General Gynecology and Gynecologic Oncology, Comprehensive Cancer Center, Gynecologic Cancer Unit, Medical University of Vienna, Vienna, Austria; 3 Karl Landsteiner Institute for General Gynecology and Experimental Gynecologic Oncology, Vienna, Austria; Central South University, CHINA

## Abstract

**Objective:**

To determine the association between isolated mid-trimester short fetal femur length and adverse perinatal outcome.

**Methods:**

This is a retrospective cohort study of patients with singleton gestations routinely assessed by second trimester ultrasound examination during 2006-2013. A fetal isolated short femur was defined as a femur length (FL) below the 5^th^ percentile in a fetus with an abdominal circumference greater than the 10^th^ percentile. Cases of aneuploidy, skeletal dysplasia and major anomalies were excluded. Primary outcomes of interest included the risk of small for gestational age neonates, low birth weight and preterm birth (PTB). Secondary outcome parameters were a 5-min Apgar score less than 7 and a neonatal intensive care unit admission. A control group of 200 fetuses with FL ≥ 5th percentile was used to compare primary and secondary outcome parameters within both groups. Chi-square and Student’s t-tests were used where appropriate.

**Results:**

Out of 608 eligible patients with a short FL, 117 met the inclusion criteria. Isolated short FL was associated with an increased risk for small for gestational age (19.7% versus 8.0%, *p* = 0.002) neonates, low birth weight (23.9% versus 8.5%, *p*<0.001), PTB (19.7% versus 6.0%, *p*<0.001) and neonatal intensive care unit admissions (13.7% versus 3.5%, *p* = 0.001). The incidence of a 5-min Apgar score less than 7 was similar in both groups.

**Conclusion:**

Isolated short FL is associated with a subsequent delivery of small for gestational age and Low birth weight neonates as well as an increased risk for PTB. This information should be considered when counseling patients after mid-trimester isolated short FL is diagnosed.

## Introduction

The fetal femur length (FL) is routinely measured to assess fetal growth during the second- and third trimester ultrasound (US) examination. The detection of a femur length below the 5^th^ percentile is often a diagnostic dilemma since this sonographic finding has been described as normal variant in constitutionally small fetuses [1–2], but may also be associated with skeletal dysplasia [3–4] or chromosomal abnormalities [5], especially trisomy 21 [6–8]. Recent studies have also suggested an increased incidence of adverse perinatal outcome such as small for gestational age (SGA) neonates [5, 9–11], low birth weight (LBW) [9, 11–12] and preterm birth (PTB) [5, 9] because of a suspected association of short FL with placental insufficiency.

Our objective therefore was to determine the association of isolated short FL detected on second trimester ultrasound (US) examination and the subsequent development of adverse perinatal outcome in a low risk patient population including only fetuses without chromosomal abnormalities and associated major congenital anomalies using a robust, prospectively obtained perinatal database.

## Materials and Methods

This is a retrospective cohort study of patients, diagnosed with a short FL, who received second trimester ultrasonography (16–24 weeks of gestation) at our tertiary referral center between 2006 and 2013. Data were assessed retrospectively using the institutions electronic documentation system and patients’ charts. Privacy of medical records were maintained according to the ICH Harmonized Tripartite Guideline for Good Clinical Practice and the guidelines of the Ethics Committee of the Medical University of Vienna. Each member of the study personnel committed to these guidelines. Patients were assigned to consecutive identification numbers and made anonymous after data collection was completed. Data was saved to the department’s password protected server and analyzed anonymously. The responsible investigators ensured that the study was conducted in agreement with either the Declaration of Helsinki and the laws and regulations of the country, which ever provides the greatest protection of the patient. The protocol has been written, and the study was conducted according to the ICH Harmonized Tripartite Guideline for Good Clinical Practice. The protocol was approved by the Ethics Committee of the Medical University of Vienna (ECS 1011–2015).

Included were only singleton fetuses of Caucasian origin. Given the known risk of adverse pregnancy outcome in fetuses with chromosomal anomalies and severe malformations, all cases of aneuploidy, skeletal dysplasia and other major congenital anomalies confirmed with postnatal examination were excluded. Pregnancies were dated by the last menstrual period when reliable or crown-rump length when dates where unreliable. Isolated short FL was defined as FL below the 5^th^ percentile for gestational age in a fetus with an abdominal circumference (AC) more than the 10^th^ percentile at the time of the ultrasound examination. All gestational age-specific biometry values were determined by standards derived by Hadlock [13]. When a short fetal femur was suspected, a detailed second trimester fetal anomaly scan was undertaken according to routine clinical practice and the patients were offered fetal karyotyping. Doppler flow indices of the uterine and the umbilical artery were performed in all cases at 20–25 weeks of gestation. Doppler examination was performed by the Pulsatility index (PI) of the umbilical artery and the Resistence Index (RI) of the uterine artery. Additionally notch evaluation of the uterine arteries was obtained. All ultrasound examinations were performed by physicians certified in obstetric ultrasound and all measurements were performed in accordance to the guidelines of the International Society for Ultrasound in Obstetrics and Gynecology (ISUOG) (www.isuog.org). The PI and the RI were considered abnormal when Doppler measurements were at the 95^th^ percentile or in the presence of diastolic notches in case of uterine artery.

Our primary outcomes of interest included the risk of SGA, defined as birth weight (BW) less than the 10th percentile, LBW (<2500g) and PTB (< 28^th^, <32^nd^, <34^th^ and <37^th^ gestational weeks). Secondary outcomes included the risk of intrauterine fetal death (IUFD), defined as fetal death at 20 weeks or more of gestation, a 5-min Apgar score < 7 and the incidence of neonatal intensive care unit (NICU) admissions. Maternal demographic information, obstetrical and medical history, maternal co-morbidities, ultrasonographic findings, genetic results, pregnancy complications, and neonatal outcomes were extracted from our perinatal database. Preeclampsia was defined as blood pressure of ≥ 140mm Hg systolic and/or 90mm Hg diastolic on two or more occasions with proteinuria of 300mg or more in 24-hour urine specimen occurring after 20 weeks gestation in a woman with previously normal blood pressure. The institution’s perinatal database was used to identify 200 consecutive pregnancies with a femur length ≥ 5^th^ percentile that were seen during the same time period at our institution’s outpatient clinic. All baseline characteristics as well as the incidence of the primary and secondary outcomes were compared between patients with and without a femur length below the 5^th^ percentile.

### Statistical analysis

In this cohort study we used descriptive statistics for analyses of patients’ demographic data. Values are given as mean (standard deviation (SD)) when normally distributed or as median (range) at presence of skewed distribution. Student’s t-test was used to compare continuous variables, and chi square test and Fisher’s exact test was used to compare categorial variables. The statistical software PASW Statistics version 18.0 (SPSS, Chicago, IL, USA) was used for statistical analyses. P-values of <0.05 were considered statistically significant.

## Results

Our study cohort consisted of 608 patients. After excluding patients with aneuploidy, skeletal dysplasia, major congenital anomalies and incomplete pregnancy data, 117 cases of isolated short FL < 5^th^ percentile with an AC > 10^th^ percentile remained for analysis. The mean (standard deviation (SD)) gestational age at the time of diagnosis was 22.4 (2.4) weeks. The mean (SD) FL at the time of the diagnosis was 33.7 (6.7) mm and the mean (SD) AC was 166.1 (29.3) mm. The demographic characteristics of the patients with a FL ≥ 5^th^ percentile were similar to patients with a FL < 5^th^ percentile in terms of maternal age, gravida, parity, maternal weight and height, body mass index, smoking status and the incidence of gestational diabetes, chronic hypertension and preeclampsia (Table 1). Fetuses with isolated short FL were delivered earlier (*p* = 0.002) and had a lower birth weight (*p*<0.001). Patients with isolated short FL were more likely to deliver SGA neonates (*p* = 0.002).

**Table 1:** Maternal demographic and clinical characteristics of patients diagnosed with an isolated short femur compared with controls.

	FL < 5th percentile (n = 117)		FL ≥ 5th percentile (n = 200)		
	N or mean	% or SD	N or mean	% or SD	P- Value
**Maternal age (years)**	30.3	5.9	29.21	6.5	0.132^1^
**Gravida**	2.4	1.6	2.8	1.7	0.0051^1^
**Parity**	1.2	1.4	1.1	1.1	0.234^1^
**Maternal weight (grams)**	63.9	15.3	65.4	15.9	0.428^1^
**Maternal height (cm)**	163.9	6.9	164.4	6.6	0.455^1^
**BMI (kg/m** ^**2**^ **)**	23.9	5.4	25.1	8.5	0.189^1^
**Diabetes**	7	6.0%	7	3.5%	0.299^2^
**Hypertension**	5	4.3%	8	4.0%	0.906^2^
**Preeclampsia**	8	6.8%	12	6.0%	0.767^2^
**Smoking**	33	28.2%	39	19.5%	0.074^2^

^1^Student’s t-test

^2^Chi-square test

SD: standard deviation, BMI: body mass index, FL: femur length

Of the 23 cases of SGA neonates 3 patients showed a history of an SGA infant. In addition we investigated the influence of a history of an SGA neonate on the rate of SGA neonates in our isolated short FL group. Women with a previous SGA infant did not show a higher rate of SGA neonates than patients without a history of SGA 19.0% versus 25% (*p* = 0.702).PTB < 32 (*p* = 0.002), < 34 (*p* = 0.003) and < 37 (*p*<0.001) weeks of gestation as well as LBW infants (*p*<0,001) and NICU admissions (*p* = 0.001) were more frequent in the isolated short FL group. We observed 16 cases of NICU admissions. In 13 cases the fetuses had to admitted because of prematurity and in three cases the term neonates suffered from transient postnatal distress. Isolated short FL was not associated with a delivery < 28 weeks of gestation or a higher rate of postnatal 5-min Apgar scores < 7. The cesarean section rate was similar in both groups (Table 2). The SGA cases (n = 23) were born at a mean (SD) gestational age of 37.7 (3.3) weeks with a mean (SD) birth weight of 2168.0 (571.1) grams. Eight fetuses in the SGA group were delivered prematurely, 3 patients because of placental abruption at 30, 35 and 36 weeks of gestation, respectively. Two fetuses had to be delivered because of signs of placental insufficiency at 30 and 34 weeks of gestation, respectively. The remaining three fetuses were delivered because of premature rupture of membranes. Doppler ultrasound studies of the uterine arteries were abnormal in 2 cases, Doppler ultrasound studies of the umbilical artery was abnormal in 4 cases in the SGA group; One of the 23 SGA cases developed preeclampsia. Both groups showed similar rates of abnormal Doppler studies of the uterine arteries, 17.9% in the short FL group versus 13.5% in the control group (*p* = 0.268), respectively. Additionally we studied similar rates in terms of the presence of diastolic notches of the uterine arteries in the two groups with an incidence of 6.0% versus 2.0% (*p* = 0.062), respectively. However fetuses with an isolated short FL were at a significantly higher risk of developing abnormal umbilical cord Doppler measurements, 5.1% (6/117) versus 0.5% (1/200) (*p* = 0,007), respectively. Four out of 6 cases with abnormal umbilical artery Doppler measurements in the short FL group were born before 37 weeks, at 30, 30, 35 and 36 weeks of gestation, respectively. Preterm birth was caused by placental abruption in two cases, one patient suffered from premature rupture of membranes and one patient underwent secondary cesarean section because of severe growth retardation and contractions. Intrauterine fetal death occurred in one fetus. Beside the diagnosis of an isolated short FL the pregnancy was normal and the routine US examinations until 38 weeks of gestation revealed no other pathologic findings. At 37+1 weeks of gestation the patient presented at the labor ward because of reduced fetal movements. The US examination revealed an intrauterine fetal death. Autopsy showed an appropriate for gestational age male neonate without other pathologic findings than the isolated short FL.

**Table 2:** Perinatal outcome in fetuses with isolated short femur length compared with controls.

	FL < 5th percentile (n = 117)		FL ≥ 5th percentile (n = 200)		
	N or mean	% or SD	N or mean	% or SD	P- Value
**Gestational age at delivery**	38.4	3.4	39.3	2.1	0.002^1^
< 28 weeks	2	1.7%	1	0.5%	0.283^2^
< 32 weeks	10	8.5%	3	1.5%	0.002^2^
< 34 weeks	11	9.4%	4	2.0%	0.003^2^
< 37 weeks	23	19.7%	12	6.0%	<0.001^2^
**Birth weight (grams)**	2785.9	752.4	3185.4	583.5	<0.001^1^
**SGA**	23	19.7%	16	8.0%	0.002^2^
**LBW**	28	23.9%	17	8.5%	<0.001^2^
Umbilical cord blood Doppler measurements ≥95^th^ Percentile	6	5.1%	1	0.5%	0,007^2^
**Gender**					
Female	51	43.6%	95	47.5%	0.500^2^
Male	66	56.4%	105	52.5%	
**5-min Apgar score < 7**	2	1.7%	2	1.0%	0.585^2^
**NICU admission**	16	13.7%	7	3.5%	0.001^2^
**Cesarean section**	60	51.3%	88	44.0%	0.210^2^

^1^Student’s t-test

^2^Chi-square test

SD: standard deviation, SGA: small for gestational age, LBW: low birth weight, NICU: neonatal intensive care unit

## Discussion

The aim of the present study was to investigate the association between an isolated short fetal FL, diagnosed at the second trimester US scan and adverse perinatal outcome. We used a strict definition of isolated short FL by excluding all fetuses with an AC < 10^th^ percentile. Additionally we excluded all cases with chromosomal and structural abnormalities to be able to reliably evaluate the effect of only isolated short FL on perinatal outcome. In this study a short fetal FL was significantly associated with a subsequent delivery of SGA and LBW neonates, as well as PTB < 32, < 34, < 37 weeks of gestation and the rate of NICU admissions after delivery. There was no association with a delivery < 28 weeks of gestation and the incidence of 5-min Apgar score values < 7. A few recent studies investigated the short FL as a feature of fetal growth restriction and confirmed the association of isolated short FL in the second trimester with an increased risk of delivering an SGA or LBW neonate [1, 3, 9–11]. Todros et al reviewed 86 consecutive referrals for femur length below the 10^th^ percentile and investigated that 21% of the patients delivered structurally normal, euploid SGA neonates. Additionally the group determined that the diagnosis of SGA was made about 9 weeks after the finding of a short femur [3]. Weisz et al, Ventura et al and Goetzinger et al similarly demonstrated an increased risk of fetal growth retardation, showing SGA rates of 19% [1], 19.7% [11] and 21.5% [10], respectively. A recent large study of 312 fetuses with a short FL, which included only cases with an AC > 10^th^ percentile showed an incidence for SGA of 13.8% and a rate of almost 19% for a birth weight < 2500 grams [9]. Our study supports the finding of about 20% SGA neonates in cases with isolated short FL. We report even a higher incidence of neonates with a LBW than in the study of Özlü et al [9] with an incidence of 23.9%. In terms of PTB the literature reveals conflicting results. Consistent with our findings, two recent large cohort studies report a significant increase in PTB < 34 and < 37 gestational weeks [9–10]. However both reports did not study the association of isolated short FL with PTB in indicated or spontaneous PTB separately. In our population we studied no significant difference in the incidence of cesarean deliveries between the cohort of short FL and the control group, drawing the conclusion that isolated short FL is a risk factor for PTB, independent of the delivery mode. On the other hand no difference in the incidence of PTB was observed in a smaller study by Weisz et al, which included only 58 isolated short femur cases [1]. Likewise, Ventura and coworkers reported that the fetuses with short FL delivered significantly earlier (38 vs 39 weeks, p = 0.01), but they did not study an increased risk of PTB [11].

The etiology of femur shortening in SGA fetuses is still unclear. However, there is a known association between abnormal placentation and fetal growth restriction. A study of SGA fetuses reported a concordance of a reduced femur length with a small AC [14], which might be explained by redistribution of blood due to chronic hypoxia, with increased flow to the heart and brain and decreased flow to the lower body [15–16]. Early identification of SGA fetuses is crucial as it is possible to decrease the risk of adverse neonatal outcome by four fold with a structured program of surveillance and accurate delivery [17]. Placental function evaluation by uterine and umbilical artery Doppler velocimetry is the clinical standard for identifying early-onset intrauterine growth restriction [18–19]. Todros et al showed an incidence of 61% of abnormal Doppler findings in pregnancies with a short FL, indicating a vascular origin of the growth disorder [3]. Similarly, in the study of Papageorghiou et al 46% of the isolated short FL cases required delivery < 37 weeks of gestation because of severe intrauterine growth retardation (IUGR) associated with abnormal Doppler studies [20]. The study of Vermeer and coworkers also reported an association between IUGR and abnormal Doppler measurements of the uterine and umbilical arteries, drawing the conclusion that Doppler studies might help to differentiate between constitutionally small fetuses and those at risk for growth retardation [12]. Our cohort of fetuses with short FL showed a significant higher amount of abnormal Doppler studies of the umbilical artery. In this group we observed 4 cases of SGA, requiring preterm delivery. Interestingly, the cause of PTB was placental abruption in half of the cases. Unfortunately our study lacks continous data about amniotic fluid values and serial Doppler findings, which clearly reflects a limitation of our study, since a differentiation between constitutional and pathological SGA, including abnormal Doppler findings and oligohydroamnios is not possible. Additionally, as our study was not designed to evaluate the timing of growth retardation in fetuses with a short FL, it is not possible to draw definitive conclusions if serial growth assessments including Doppler studies may be warranted in these cases.

As placental insufficiency is the most common cause of growth retardation it seems likely that IUGR and short FL might be linked to hypertensive disorders and preeclampsia. Zalel et al observed that seven out of nine patients with an isolated short FL developed hypertension [21] and Todros et al observed a 19% incidence of preeclampsia in structurally normal fetuses with a short FL [3]. Our study did not show a difference in the incidence of hypertensive disorders or preeclampsia between the two groups, which might be explained by the small number of cases. Consistent with our findings, the recent large cohort studies have demonstrated no statistically significant increased risk of preeclampsia in patients with an isolated short FL [9–10].

Beside chronic hypoxia as a cause of short bones, two other mechanisms were suggested for the etiology of short FL. Zalel and Mancilla et al proposed altered secretion of fibroblast growth factor 2 (FGF-2) due to abnormal placentation, resulting in inhibition of long bone growth [21–22]. Additionally other studies could show that maternal serum Pregnancy-associated plasma protein A (PAPP-A) levels measured at 10–14 weeks of gestation are significantly associated with the length of fetal long bones [1, 23–24], which can be explained by the fact that PAPP-A regulates osteoblast proliferation by increasing the bioavailability of insuline-like growth factor-binding-protein-4 [25]. Unfortunately, due to the retrospective design of our study, we lack data about PAPP-A, which seems to be a promising prognostic parameter for the FL in the first [23] and second trimester [24]. Further studies are needed to test these hypotheses.

Some authors have described the different ethnic origins in their study population as a study limitation [1, 10] and recommended ethnic specific femur length growth charts [9]. All of the patients in our study, both in the short femur and normal femur length group were of Caucasian origin, which may reflect a strength of our study. A limitation is clearly the retrospective study design as well as the relatively small number of cases, compared to the recent large studies by Özlü et al and Goetzinger et al [9–10]. Additionally we did not adjust for maternal height and weight, since we observed no difference in these parameters between the case and the control group as both were of the same ethnic origin.

## Conclusions

In conclusion this study shows a significant association between the finding of an isolated short FL at the second trimester US examination with SGA and LBW neonates and PTB. However it is important to clarify that the prospective parents can expect a term delivery of a healthy, appropriate for gestational age neonate in 73.5%. Abnormal Doppler measurements of the uterine and umbilical arteries may help to differentiate between fetuses with a constitutionally small FL and those at risk for SGA. Larger studies are needed to determine the performance of a reliable screening protocol by the combination of serial US examinations, Doppler studies of the uterine and umbilical arteries and biomarkers such as PAPP-A.

## Supporting Information

S1 Dataset Contains all relevant data and was provided upon submission. (XLSX)
